# Family context and individual situation of teens before, during and after pregnancy in Mexico City

**DOI:** 10.1186/s12884-017-1570-7

**Published:** 2017-11-16

**Authors:** Reyna Sámano, Hugo Martínez-Rojano, David Robichaux, Ana Lilia Rodríguez-Ventura, Bernarda Sánchez-Jiménez, Maria de la Luz Hoyuela, Estela Godínez, Selene Segovia

**Affiliations:** 1 Departamento de Nutrición y Bioprogramación, Instituto Nacional de Perinatología, Mexico City, Mexico; 2 Departamento de Posgrado e Investigación, Escuela Superior de Medicina del Instituto Politécnico Nacional, Plan de San Luis y Díaz Mirón s/n,Colonia Casco de Santo Tomas, Delegación Miguel Hidalgo, 11340 Ciudad de México, Mexico; 3 Posgrado en Antropología Social, Departamento de Ciencias Sociales, Universidad Iberoamericana, Mexico City, Mexico; 4 Universidad del Valle de México, Mexico City, Mexico; 5 Centro Nacional para la Salud de la Infancia y la Adolescencia, Secretaría de Salud, Mexico City, Mexico

**Keywords:** Teen pregnancy, Adolescents, Self-esteem, Qualitative research, Family, Mexico

## Abstract

**Background:**

In the last 20 years, adolescent pregnancy has become one of the most critical problems affecting women in Latin America and the Caribbean.

**Methods:**

This qualitative study was based on in-depth interviews with 29 teen mothers. All of the pregnant teens were from low- to lower-middle-class social strata in the Mexico City metropolitan area. The family (living with the girl) and the individual context of pregnant teens were analysed on the basis of data from at least three interviews: during pregnancy and at approximately 6 and 24 months following delivery. Additionally, six mothers, four fathers, and four partners of the pregnant girls of the group were interviewed. The information on the individual and family situation before, during and after the pregnancy was recorded and transcribed, then analysed in three phases, comprising pre-analysis, exploration and interpretation.

**Results:**

The pregnant teens had a family background of teen pregnancy. The girls disclosed feelings of repression, loneliness and indifference to their parents, leading them to unprotected sexual relations without fear of pregnancy. After the pregnancy, communication improved between the girls and their parents, but became worse with their partner. Consequently, these teens returned to feeling as they did before getting pregnant. They stated that they would make their situation work for the sake of their child, and regretted dropping out of school and getting pregnant so young. Almost all said they were seeking love outside the family, which revealed a scenario of limited communication and unsatisfactory relations within the family.

**Conclusions:**

Understanding how communication works between parents and children is necessary to avoid teenage pregnancy, as well as early marriage or cohabitation, resulting in dropping out of school and financial constraints, which lead to great frustrations between the couple and affects the child. In addition, it is vitally important that adolescents be motivated in the family setting in order for them to continue their studies. There is also an urgent need to implement measures that compensate for educational inequality, as well as to strengthen strategies aimed at adolescent mothers and pregnant teens that encourage their school performance through the support of scholarship programs and day care centres. Many of the problems inherent in adolescence are related to the lack of affection and support, and in many cases are a reaction to authoritarian rules or limits established unilaterally by parents with little or no dialogue involved.

**Electronic supplementary material:**

The online version of this article (10.1186/s12884-017-1570-7) contains supplementary material, which is available to authorized users.

## Background

In the last 20 years, pregnancy among adolescents has become one of the most critical problems affecting women in Latin America and the Caribbean. Pregnancy in adolescence carries greater medical and psychosocial risks, which generate problems in public health, justice and education; the risk of maternal death is four times higher in adolescents younger than 16 years [1]. In addition, among adolescents 15 to 19 years of age, pregnancy-related death is the second leading cause of death after accidents. Younger mothers are at increased risk of developing obstetric fistula, anaemia, eclampsia, postpartum haemorrhage, and puerperal endometritis. Also, adolescents younger than 19 years of age have a 50% higher risk of stillbirths and neonatal deaths, as well as an increased risk of developing preterm birth, and having newborns with low birth weight and asphyxia. Besides affecting maternal health, early marriage and maternity also prevent adolescents from attending school and perpetuate the cycle of poverty and ignorance [1, 2].

On the other hand, it is important to emphasize that teenage motherhood is often considered a step toward adulthood and offers an improved status within the community [3]. Becoming a mother is a way to conquer new respect and become a complete woman as defined by the surrounding society. Adolescent motherhood is seen as an option that gives meaning and purpose to life, particularly in contexts where there are few or no alternatives. However, it is important to note the frequent relationship between sexual violence and pregnancy in adolescence, particularly in the case of adolescents under 15 years of age [3].

Although there has been a decline in fertility in the Latin American region, data on adolescents who have unwanted pregnancies show that they are no longer considered subjects with rights; their ability to make decisions regarding their bodies is denied, and their access to health services, information and sex education is restricted. The high rates of teenage pregnancies are linked to the problem of forced marriages, sexual abuse and violence, and the prohibition of voluntary abortion [4].

Moreover, in the developing countries, one in three women between the ages of 20 and 24 married before the age of 18 (70 million women), and a third of these married before the age of 15 [5]. United Nations Children’s Fund (UNICEF) in 2015 reported that 30% of women aged 20–49 in Latin America and the Caribbean were married before the age of 18, while 18% were married before the age of 15 [6]. In Mexico, according to the National Demographic Dynamics Survey in 2014, it was reported that 21.5% of women aged 20–24 were married before age 18 and 3.8% before the age of 15. Currently, one in five women were married or in a free union before reaching the age of majority (18 years old) [3]. Adolescent marriage rates in Latin America and the Caribbean are somewhat lower than the rest of world. Nonetheless, while the practice of child marriage has been declining slowly since the 1980s, there have been no noteworthy changes in Latin America and the Caribbean [6].

In addition, in Latin America, the prevalence of child marriage in rural areas is almost double that of urban areas; adolescents from poor families and those living in rural areas are particularly vulnerable. Whereas in the wealthiest quintile it is estimated that 10% of women aged between 20 and 49 married before the age of 18, the proportion is as high as 38% for the poorest quintile. Indigenous adolescents are particularly affected, which contributes to the reproduction of intergenerational poverty [7]. However, unlike rural and urban areas in Mexico, conjugal unions do not occur especially early in rural zones compared with cites; in other words, child marriage is not a problem that exclusively affects girls or adolescents in rural areas [8].

There has been a marked, although uneven, decrease in the birth rates among adolescent girls since 1990, but some 11% of all births worldwide are still to girls aged 15 to 19 years old. The vast majority of these births (95%) occur in low- and middle-income countries. The 2014 World Health Statistics indicate that the average global birth rate among 15 to 19 year olds is 49 per 1000 girls. Country rates range from 1 to 299 births per 1000 girls, with the highest rates in sub-Saharan Africa. Complications during pregnancy and childbirth are the second leading cause of death worldwide. Adolescent pregnancy remains a major contributor to maternal and child mortality, and to the cycle of ill-health and poverty [9].

In Mexico, the births of adolescent mothers have increased. In the year 2000 births for women between 15 and 19 years represented 15.7% of the total and in 2013 this proportion was 16.3%. The highest rates are: Coahuila (86.3), Chihuahua (84.9) and Sonora (83), while Mexico City, Guanajuato and Querétaro have lower rates: 49.2, 56.9 and 57.3 births, per thousand adolescents, respectively. The 2011 fertility rate for women aged 12–19 was 37 births per 1000 women. In 2013 there were 77 births per 1000 adolescents between the ages of 15 and 19. Of all adolescent women (12 to 19 years of age) who had sex, half (51.9%) had been pregnant and 10.7% were pregnant [9]. On the other hand, unsafe abortions vary substantially by age across regions: adolescents (15–19 years) account for 25% of all unsafe abortions in Africa, whereas the percentage in Asia, Latin America, and the Caribbean is much lower. By contrast, 42 and 33% of all unsafe abortions in Asia and Latin America, respectively, are in women aged 30–44 years, compared with 23% in Africa. For the developing regions as a whole, unsafe abortions peak in women aged 20–29 years. On the basis of World Health Organization (WHO) estimates, if current rates prevail throughout women’s reproductive lifetimes, women in the developing world will have an average of about one unsafe abortion by age 45 years [10].

Teenage pregnancy has not diminished in Latin America and the Caribbean, despite great efforts in public policy for that purpose [11]. The predominant reason for this ongoing problem, according to the literature, is an established family pattern [12]. Whereas in some societies the event of a teen pregnancy is mainly seen as a limitation, including the economic burden and truncated or restricted opportunities for professional development [13], in other social contexts it is representative of fertility [14] and adult status and leads to recognition by the community [15].

Other factors related to teen pregnancy are low self-esteem, feelings of abandonment and loneliness, and poor communication between the girl and her mother regarding the onset of sexual relations [16]. The latter element can have a very negative influence even when the teen girl has adequate information about birth control [17].

Accordingly, a teen pregnancy can derive from a combination of factors in the family setting, including economic and psychological aspects. This means that the causes of this predicament are particular to each case and as yet uncertain [18]. Moreover, this phenomenon is not exclusive to one type of family or to a particular kind of neighbourhood. Finally, most pregnant teens receive support from their mother and partner [19].

The present qualitative study explores the social reality of pregnant teens in the Mexico City metropolitan area. The data was gathered from interviews with the girls and key informants around them, and thus represent their own perspective and family context in relation to their situation before, during and after pregnancy. The aim of the study was to explore factors in the individual and family context of teenage girls that can be present with teen pregnancy.

## Methods

### Design

This qualitative study was carried out during and after the pregnancy of a group of teen mothers in Mexico City. Data gathering was based on about 90 in-depth interviews with 29 teen girls, and key informants carried out by the first author of the working group.

Information on the life history was gathered by talking with the adolescents [20]. The ethnographic tool of interviewing with an observer present reduced the incidence of reactivity and of acting differently during the interview, allowing the researcher to get accustomed to the participants and their families and vice versa. This facilitated a mutual involvement in the activities and conversations [21] and a better understanding of the context of adolescent pregnancy was gained, based on the relationships achieved over a period of more than 4 years of fieldwork, marked by the cooperation and availability of the interviewer and informants. Objectivity was achieved and maintained respecting the participants’ dialogues in order to reduce bias and control the effects of the researcher on the research situation. To achieve this, a considerable degree of interaction was maintained with the participants, based on participant observation.

### Interview

Prior to the interviews, an interview guide was developed and approved by three specialists in the fields of social anthropology and adolescent psychology. The interview guide was piloted with a pregnant teen and a teen mother, as well as with a teen mother and a relative of the latter contacted through a nurse. The comments of the working group and specialists thus enabled the guide to be improved.

The interview guide included possible related aspects of the family and individual (personal) context of the adolescents before pregnancy such as length of the relationship, initiation of sexual relations, relationship with the family, their emotions and prospects for the future, and reasons why they decided to get pregnant. Regarding pregnancy, the questions centred on the reactions and responses to news of the pregnancy on the part of the parents, brothers and boyfriends of the adolescent. Questions regarding the period after delivery centred on their emotions and prospects for the future, relationship with the family, boyfriend/husband/partner, and self-perception of being a mother. The interview guide can be consulted in the section of Additional file 1, titled “Interview guide for pregnant teens”.

### Participants

The girls and key informants were residents of the Mexico City metropolitan area, and the pregnancy occurred between 2008 and 2013. The heads of household worked mainly as street vendors (in the informal economy at their own street stands), servants, construction labourers, carpenters, plumbers and as farmers. The average level of education of the head of household was the completion of middle school (9 years of formal education), and the socioeconomic level was low- to lower-middle class. The pregnant teens, with an average age of 15.4 years, had sought prenatal care at a public health centre. The average age of their partner or spouse was 20 years. Among the characteristics of the participants, it was observed that all had a background of blood relatives or in-laws with teen pregnancies. Regarding birth order, 6 of the 29 cases were the oldest child, 10 were the middle child, 11 were the youngest child and two were only children.

### Sampling

The sample was consecutive and intentional, making contact with women having had pregnancies without complications before age 19; no cases of pregnancy due to rape or sexual abuse were included in the sample. The snowball technique was used to obtain new cases, contacting teens at school, in their homes, or in the clinic during prenatal care. The adolescents were approached and invited to participate in the research. The search for participants and the interviewing process were halted when we observed a saturation of information obtained in relation to the subjects of interest. This point was reached with 29 interviewees with about three interviews per adolescent with a follow-up duration of approximately 2 years from the first contact. As most adolescents have their first prenatal check-up in the last trimester of pregnancy, the experts and the working group decided to perform only one interview during pregnancy to explore contents prior to and during the pregnancy. The aim of other two post-partum interviews was to obtain information about the pregnancy, and individual and family contexts after the pregnancy.

### Data gathering and generation

The interviews were semi-structured and conducted face-to-face with each of the teen girls one at a time. All interviews with any given key informant were carried out by the one researcher-interviewer who had not had any relationship with the interviewee. The 29 pregnant teens were each interviewed at three different moments: during pregnancy and at approximately 6 and 24 months following delivery. In order to understand the dynamic of each individual process and to explore what occurred over time, we chose a period of 2 years to see what had happened after childbirth in the areas of family relations, school, partner relationships and future projects.

Additionally, six mothers, four fathers, and four partners of the pregnant girls of the group were interviewed. Since the context of teen pregnancy should be considered as an integrated process, we wanted to fully explore its factors from the perspective of the family. Accordingly, in addition to the information provided by the teen interviewee, we sought out the opinions of relatives or people close to her. With these varied sources, we sought to complement and enrich the information, and to gain knowledge of the testimony of the family members, thus qualifying it for the individual and family context of teen pregnancy.

The participants were allowed to establish the places and dates of the interviews in order to attain a better flow of information, resulting in that homes and parks were the most frequent settings. An anthropologist and an observer (researcher) conducted the interviews. They were recorded and lasted an average of 45 to 50 min; the researchers in parallel with the interview process subsequently transcribed the recording. To ensure methodological rigor, an independent social psychologist and two anthropologists participating in the working group listened to the recordings and read over the transcripts several times. The aim here was to provide an objective dimension to the data obtained and reach a consensus on the research issues. It also provided us with a space for reflection so as to better proceed with the subsequent phase.

### Ethical considerations

Each participant and their parents or guardians signed the letters of assent and informed consent, guaranteeing anonymity the confidentiality of their data. In the case of adolescents over 18 years of age, only informed consent was requested as under Mexican law, they are considered legally competent to make decisions regarding their participation in the study. The protocol was approved by the research and ethics committees of the National Institute of Perinatology and all participants were offered free nutritional care in turn for their participation. Information leaflets providing nutritional guidance and information on healthy eating habits, following the guidelines set forth in the Ministry of Health’s Official Mexican Standard, NOM-043-SSA2–2005, were developed for and distributed to all participants in the present research.

### Data analysis

In order to obtain the best possible interpretation, the information was analysed in its entirety in three phases: pre-analysis, exploration and interpretation of the results [22]. In the pre-analysis phase, some authors read the transcribed interviews and four of the authors independently listened to the recordings, which was their first contact with the context. Subsequently, as a group, the texts that had been previously read were discussed and the information codes of each of the parts of the interview guide were selected, allowing us to organize and analyse the data as an integral process centred around the teen pregnancy event. Here it was determined that the same codes would have to be analysed in the different interviews to be subsequently carried out so as to examine the teen pregnancy as a dynamic process. The exploration phase consisted of developing coding categories centred on the information from the interviews and the pertinent literature consulted, in which mutual exclusion, homogeneity and belonging were considered, in other words, in line with the intent of examining the dynamic of the same categories before, during and after the pregnancy. These included emotions, reactions of family members and relatives to news of the pregnancy, relations with family members, the partner or boyfriend, perspectives for the future and self-perception as a mother.

The categories of the analysis were established according to the adolescents’ own approach to their experience during and after pregnancy in order to get a better grasp of their family and personal context. The aim here was to succeed in interpreting the data through the social construction of the experiences of participants during pregnancy, motherhood and their married life. In general, the idea was to gain a clear understanding of the context of pregnancy as a dynamic process, focusing on different situations, in order to move from the particular to the general, and to be able to contextualize the group of adolescents interviewed.

The information obtained from the interviews of all of the participants was used to develop some explanations of the context of teen pregnancy. To this end, the data from our research were related to those reported in other studies, always bearing in mind the conceptual framework of the pregnancy and the teenager. The purpose here was to make sense of the information provided by each of our interviews and participants before, during and after the pregnancy.

## Results

### The family context before pregnancy

An intergenerational pattern was observed with regard to teen pregnancies. Family relations were diverse and, although the nuclear family was predominant, there were also extended, uniparental and blended families. The relationship of the teen with the family was of a diverse nature. There were some instances of *poor* relations between the girl and her mother or stepfather, which were characterized by arguing, discord and little real communication within the household. For example, 16-year-old Sandra (married) commented:

*I did not want any more mistreatment or physical abuse, and that is why I left… I have two half-brothers, both younger, who were treated better than me. My stepfather hit me in front of my mother and she did not say anything.*

Other types of relationship were defined as “good family relations”, according to the teen girls, and were based on the absence of fights, even in the presence of repression. These teens had their material needs covered, but did not like it when they were prohibited from going to parties or arriving home late. This situation was described by 13-year-old Yaqui (cohabiting with her boyfriend):

*Everything was all right with my mom— she did her thing and I did mine. It is just that I was fed up because she did not let me go to parties.*

“Normal family relations” were referred to when the teen girl and their parents got along well, even though fights did occur at times. Quite notable in these cases was ambivalence in the girls’ emotions, leading to having sexual relations and pregnancy. This situation is represented by 18-year-old Jazmin (single):

*I felt very restricted… my father kept a close eye on me. Even though I did not love Pedro* [father of her child]*… I had relations with him. I did so because my parents did not like him.*

One characteristic that has been documented within the context of the pregnancy in the adolescents is dropping out of school, a situation that was confirmed with our interviews: there were cases in which the participants did not like school and before they got pregnant they had already left school, while others did so during pregnancy. On becoming pregnant, 18 teen girls dropped out of school. Another 11 had decided to start working or simply stay at home and rest. As Carmen, a 15-year-old (living with her boyfriend) reported:

*I don’t like school, I asked my parents to let me take off a year. But I did not come back and went to live with my son’s father.*

The average level of education of the teen girls was short of completing middle school (9 years), this level being similar (the same or less) to levels in the previous generation. The mothers of these girls were employed outside of the home as servants, street vendors, seamstresses or workers. Consequently, they spent time with their teen daughters only on weekends. In three cases, the mothers were widows or single and had to migrate to other cities while their daughter stayed with the grandparents.

The level of education of the girls was the same as, or lower than, that of their parents. As 15-year-old Silvia described:

*My mom and my dad studied until high school, but I did not even finish it. School was not for me.*

### Perceptions of feelings

The adolescents reported that they felt lonely and indifferent to their parents, who only intervened to scold them about normal fun activities for teenagers. This observation was independent of their place in the family hierarchy among siblings (Table 1a, b, c, d). The girls who were an only child had adequate economic support, but communication with their mothers was commonly antagonistic. As 15-year-old Rosa said:

*I was always fighting with my mother. I got along well with my dad since I almost never saw him. He leaves early and arrives home late.*

Of great importance is that 21 of the teenagers stated that they had sexual relations with the intention of seeking love. In this sense, the teenagers emphasized that their loneliness and the indifference and repression that they perceived from their mother and father led them to secretly go out with a boy, resulting in brief dating periods before sexual relations began. The encounters between the teen girl and her boyfriend generally took place when the parents and relatives were not at home, and they usually met on the street corner closest to her house. The young couple would then covertly enter the house. The boyfriends were in most cases school or work mates, and some times neighbours. However, the beginning of sexual relations was motivated by anger or curiosity for some participants. Even though the teens had the stated intention of starting their own families, this situation was not expected *so soon*. The girls stated that they had unprotected sexual relations out of feelings of love, even rejecting some form of birth control when offered by the boyfriend.

*He [her boyfriend] told me to use a condom, and I answered: “No, it’s not the same… and we want to have a family. (Lizeth, 17 years old, only child).*

When the girl began going out with a boy, the relatives noted a radical change in her behaviour. She became temperamental (often crying), aggressive, sad and indifferent, and insisted on permission to leave the house with greater frequency.

*When they begin to always ask permission to leave the house, you have to be very careful… It had already been several months that Lucero asked for permission, and very frequently, to go somewhere with her girlfriends… she was always angry and cried about everything. (Regina, 50 years old, mother of Lucero).*

**Table 1:** Narratives for analysis

Before pregnancy: perceived emotions, including lonely and indifference to parents a) *My mother tried hard to provide us with everything we needed. I never even saw her… but I would have preferred that she was close to me.* (Eréndira, 16 years old, youngest child) b) *Because I feel that, my mother was not me when I needed her, I wanted her to talk to me about my things, but she preferred to be with her boyfriend…* (Josefina, 16 years old, oldest daughter) c) *My mom always had preferred my younger sister… she used to say that “she* [the younger sister] *really studies, not like you…” With my younger sister she is very tolerant… she gives her everything.* (Vanesa, 18 years old, middle child) d) *Well, I think that the relation with them* [her parents] *has always been relegated to convenience, not based on feelings. Their words are: “Behave yourself and I won’t bother you.” They always left me with my grandfather, the father of my father.* (Tonantzin, 16 years old, only child) e) *He* [her boyfriend] *told me to use a condom, and I answered: “No, it’s not the same… and we want to have a family.* (Lizeth, 17 years old, only child) f) *When they begin to always ask permission to leave the house, you have to be very careful… It had already been several months that Lucero asked for permission, and very frequently, to go somewhere with her girlfriends… she was always angry and cried about everything.* (Regina, 50 years old, mother of Lucero)
During pregnancy: family reactions to the event, including anger, joy? and uncertainty g) *When I left home, my brother got very mad at me for leaving without finishing school. When I got pregnant, it was seen as normal since I was already living with my boyfriend.* (Marcy, 16 years old, cohabiting) h) *She asked me if I felt like having a baby and in fact from the time I was 13… 14 years old I wanted to have a child… I could not wait to be a father.* (Juan, 18 years old, boyfriend of Karen, single) i) *With him* [her boyfriend]*, what can she expect? He does not even have enough to buy food. She will stay with us! I will support her.* (Juan, 38 years old, father of Jazmin) j) *At first, my parents got angry, but then it happened and they helped me in everything I needed.* (Daniela, 16 years old, single) k) *…And he* [her brother] *told me: you are already a mother and nobody is here to support you… If you get pregnant again, you will not leave the house, not even to go to school.* (Lucero, 15 years old, single)
After childbirth: the reality of uncertainty, limited income and unfinished schooling l) *My daughter is not used to doing without, and he* [her boyfriend]*, what can he offer her? He can barely provide for himself… he does not have a steady job.* (Hugo, 41 years old, father of Chela) m) *He* [her boyfriend] *went to work, painting a garage door, cutting grass… He did not finish school. He could not handle it. He was in school, had homework, and had to work... he did not have enough time.* (Nancy, 18 years old, cohabiting) n) *I already feel somebody in my family; because now with my mother I talk more about the care of the children.* (Maria, 16 years old, cohabiting) o) *I do the house cleaning and make dinner while my mom is at work, and after returning she helps me take care of my girl, and I go to junior high.* (Karina, 13 years old, single) p) *He* [her boyfriend] *was very different. He always was waiting for me with roses or some gift… I felt very secure… he does not do that anymore.* (Gina, 16 years old, cohabiting) q) *I felt lonely before getting pregnant… my parents did not even realize what was happening with me. Now that I live with Rubén* [her husband]*, I am feeling the same thing… he is almost never with me and I prefer it that way… he is almost always angry. Only the presence of my child consoles me… for him I put up with everything.* (Sayra, 15 years old, married) r) *My son is the best thing that could have happened to me, I love him very much and he is my consolation, because sometimes I feel lonely.* (Rosario, 16 years old, single) s) *Being mother brought me satisfaction whit my daughter, but I regret not having finished school or living longer as a single, I lacked more fun.* (Pilar, 16 years old, cohabiting)

### Reactions to the pregnancy

The reactions to the pregnancy were varied. For the girls who were married or living with their partner (7/29), the news was not a surprise to either the couple or their family (Table 1g). In instances where the girl was single (22/29), the boyfriend reacted with joy and wanted the pregnancy to continue to term (Table 1h), and therefore proposed living together. Of the 29 cases, only five boys did not provide economic support to the pregnant girl. Sixteen couples started living together since the rest were not permitted to do so by their parents.

*With him [her boyfriend], what can she expect? He does not even have enough to buy food. She will stay with us! I will support her. (Juan, 38 years old, father of Jazmin).*

The usual pattern for the parents, upon hearing of the pregnancy, was to experience anger, sadness and disappointment, which identifies the cycle of feelings, including acceptance, and even the encouragement of an early marriage. As Fabiola (a 15-year-old) said:

*My mom was very angry when I ran away to get married, because my grandmother wanted me to get married the “right way”. My mother and all of my aunts got married the right way, even though some of them no longer live with the father of their children.*

In spite of their emotions, the parents provided material, emotional and practical support (Table 1j). The grounds for this decision were that they, too, were given support when in the same situation. In some instances, the support provided by the parents jeopardized the economic stability of the family because the mother decided to leave a paying job to take care of her daughter.

In the majority of pregnancies, the girl decided to start living with her boyfriend who promised marriage in the future. Yet after one or 2 years of living together, none of the couples had married, due to economic reasons. In fact, three of the teen mothers had separated from their partner before the end of the study. Sixteen girls moved several times, changing between virilocality and uxorilocality. The single mothers remained at the home of their family, thus generating much greater expenses for their parents. Uxorilocality (living with the girl’s parents/family) or virilocality (living with her partner’s parents/family) implied an important source of assistance for the new mothers. It should be clarified that among the reasons given for constant changes of residence during and after pregnancy were arguments and problems with in-laws. The girls subjectively experienced this situation in different ways, with some feeling a constraint on their freedom following the birth of their child.

*…And he [her brother] told me: you are already a mother and nobody is here to support you… If you get pregnant again, you will not leave the house, not even to go to school. (Lucero, 15 years old, single).*

Six of the girls were prevented from getting married or cohabiting, and these teens made up their minds to live with their partner once they reached the age of majority. This is the age of 18 in Mexico and underage adolescents must have the signed consent of their parents or guardian in order to legally marry.

### The new reality: relations with the partner/family; self-perception of being a mother

In cases of single mothers, the parents expressed the desire that their daughter continue studying and that she have greater material comfort than her boyfriend could provide (Table 1l). These parents asked themselves what kind of life they could expect for their daughters with a boy who had not finished high school. However, it is important to emphasize the effort made by the boys who reacted responsibly to the pregnancy and were forced to drop out of school and go to work. This was true whether they lived with the new mother or not. For these boys, there were obvious academic and economic implications of this decision.

*He [her boyfriend] went to work, painting a garage door, cutting grass… He did not finish school. He could not handle it. He was in school, had homework, and had to work... he did not have enough time. (Nancy, 18 years old, cohabiting).*

For the girls who formed a family, the context was one of seeking the attention, love and time not provided to them by their parents. This situation was expressed by Marietta, a16-year-old (single):

*With my boyfriend, I did not feel lonely and I feel love .Once I was pregnant, my parents did not let me be with him, and my mom stopped doing her things in order to take care of me. I feel they pay me more attention to me, even though I live separated from him* [her boyfriend]*.*

Becoming a mother meant fulfilment for the girls in spite of the fear and worry experienced when they did not know how to inform their parents of the pregnancy. The expectations of each of the teen girls centred on the well-being of their child. They also expressed the desire to return to school and to work in any kind of job they could get to make ends meet.

In all cases, the relation between the teenage girl and her parents improved after the pregnancy (Table 1n), with parent-daughter communication centring on the care and needs of the child. Some parents encouraged their daughter to continue her education, facilitating her return to school while they took care of the grandchild (Table 1o), an opportunity which was taken advantage of by four teenagers during the follow-up time. Although some of the teenagers in the study did not like school and preferred to work, for most of them there was no work to be found.

On the other hand, among the girls who lived with their partner upon getting pregnant (15/17), the reality of married life was not what they had expected (Table 1p). In addition, there was repeated mention of a perception of loneliness and indifference, but they preferred that life to returning with their parents.

*I felt lonely before getting pregnant… my parents did not even realize what was happening with me. Now that I live with Rubén [her husband], I am feeling the same thing… he is almost never with me and I prefer it that way… he is almost always angry. Only the presence of my child consoles me… for him I put up with everything. (Sayra, 15 years old, married).*

In some cases, this was due to the heavy workload of their partner who was away long hours of the day or even for weeks at a time. Consequently, the girls did not receive the attention that they had experienced when going out with their boyfriend before getting pregnant. For this reason, the child represented a consolation for some of the new mothers and a reason to put up with the indifference of their partner.

*My son is the best thing that could have happened to me. I love him very much and he is my consolation, because sometimes I feel lonely. (Rosario, 16 years old, single).*

Contradictory narratives were given with respect to the self-perception of the girls as mothers. Some argued that their child temporarily compensated for their solitude, while others described their indifference to the child. Still others felt their desires repressed and regretted not having continued their studies. These girls argued that they did not have fun anymore, since they were always busy taking care of their child or their partner (Table 1s).

*Being a mother brought me satisfaction with my daughter, but I regret not having finished school or living longer single. I don’t have fun anymore (Pilar, 16 years old, cohabiting).*

## Discussion

### The experience prior to pregnancy

The characteristics in the individual and family context of teenage pregnancy in our sample were diverse and varied and can be understood at different levels of analysis. A perspective is macrosocial, considering socioeconomic structure, current public policy, social class, gender and culture, such as early marriage or cohabitation and its legal framework. Another perspective is based on the individual, taking into account their knowledge, attitudes, perceptions and behaviours, as well as the family structure and social support network. Our results are in agreement with those reported by Kostrzena [23], whose study of three Latin American countries concluded that it was important to conduct research on sexual and reproductive health among adolescents. This study also covered issues in different contexts of teen pregnancy such as knowledge of sexually transmitted disease, childbirth practices, the quality of pre-natal care, the use of contraceptive methods, family planning and abortion, among other topics. It also established the importance of understanding the factors contributing to the increase of sexual risk behaviours and negative reproductive health outcomes among young people fundamental in comprehending the phenomenon and establishing public policies and strategies to reduce adolescent pregnancy. In addition, the results of the different cases analysed should be useful for informing and improving the interventions of the health services in different contexts, both family and individual. With the present study, we aim to contribute to the topic by shedding light on the sociocultural context of adolescents before, during and after pregnancy, helping to understand this health event that continues to occur frequently in countries such as Mexico.

### Importance of the family and the pursuit of love outside of it

Regarding the characteristics of the family, previous studies [24, 25] had shown that the majority of adolescents having experienced maternity or paternity suffered from the absence of a maternal or paternal figure, which resulted in a lack of attention in the family home. In our research, this absence was found to be due to demanding work, the parents’ separation, or to the death of one or both parents. The results shown in the present study are consistent with the observations made by Ellis B et al. [25], who examined adolescent sexual activity and adolescent pregnancy. Their findings noted that girls who experienced the father’s absence before age five of age had higher rates of early sexual activity and adolescent pregnancy.

In the majority of cases analysed in our study, families were extended, and were composed of 4 to 6 children on average. This family situation had an effect on the decrease in the attention that the adolescents received from their parents, which contributed to poor communication among family members, feelings of loneliness on the part of the adolescent, despite daily interaction with brothers and sisters. Unstable intrafamilial relationships, with or without physical or emotional violence, was mentioned by the teenage girls in our interviews. Our findings agree with those of Miller et al. [26], who analysed the results of two decades of research on the influence of the family and, especially the father, on the risk that their daughters’ adolescents become pregnant or that their male children impregnate other adolescent women.

However, in most of the interviews in our study, the adolescents reported that both parents worked and, in some cases they lived in a household with a mother and stepfather, resulting in a conflictive relationship that contributed to the parents paying less attention to the children. These factors concurred to give rise to an inadequate role of the paternal or maternal figure, who otherwise could have attended to, supervised and guided the adolescent girl when faced with the difficulties that normally occur at that stage of life. The opportunity was thus lost to provide the support, guidance and the love that should be given to adolescent children, as documented by Ruiz-Casares and Heymann [27]. These authors concluded that insufficient social support for working families often resulted in unsafe arrangements for the care of children and limited parental involvement in education and health care. Limited participation of parents in education and health care exposed adolescents to pregnancy and other situations such as addiction and obesity.

In the context of this attempt to deal with a complicated family environment, the love relationship between two adolescents would represent, in part, the cornerstone for building a symbolic representation of the couple and its implicit sexuality. At the same time, the young couple wants to share feelings, emotions and sense of a suitable parental environment in a space where both feel desired and valued. As our analysis of interview information indicates, both teens wanted to be parents, but not so soon. This stands in contrast to a qualitative study conducted on Chinese women [28] in which it was found that adolescent girls did not want to become mothers. That study found that pregnant teenagers took into consideration their relationship with their boyfriend, family advice or support, practical considerations, their personal values in life and opinions about adoption. We consider that the findings of our study may be due to the fact that the adolescents come from dysfunctional families, where they have not found love or support. In the face of the opportunity to start a new family, they are excited to feel loved and to be loved, which offers them with the possibility of leaving a dysfunctional family environment.

In studies conducted in Latin America and the Caribbean, it has been well documented that a family history of pregnancy during adolescence is a predictor of the same event in the current generation [11]. This phenomenon, in fact, was observed in the present study, evidenced by a family history of adolescent pregnancy in the majority of the cases (among blood relatives or relatives of the previous generations). This matches findings by East et al. [29] in their analysis of 127 Latin women in which in-depth interviews revealed that the young women whose sisters and mothers had given birth during adolescence had a greater probability of becoming adolescent mothers themselves. Thus, adolescent pregnancy in the new generations is more frequent where there is a family history of adolescent pregnancy and where the socioeconomic and family environment has not changed for generations. In such contexts, legal and common-law marriage, as well as pregnancy during adolescence, are not considered to be problematic.

The main contradiction in the concept of seeking love is the emotional instability of adolescents, mainly in their behaviour during the short period of courtship. The teens referred to being in love and that they had not wanted to get married or get pregnant so soon. However, they rejected the use of contraceptives, some with the argument of wanting to be mothers, a reasoning that has been documented in other studies [30]. The same research showed that adolescents who reported that their sexual relations were exclusively romantic shared their emotions more intensively with their partners. It is probably for this reason that the adolescents did not inform their family of the relationship with their boyfriend.

Dreams and fantasies play an important role in the lives of adolescents. They constitute an element of expression of sexuality and are frequent and explicit. Fantasy accomplishes different functions: it substitutes real but inaccessible or feared experiences, it serves to prepare for a real relationship later on in life, it allows adolescents to gradually recognize their sexual responses and, in general, it is a form of experiencing sexuality without risks and in a controlled manner. From their fantasies, many adolescents get involved in relationships that at the same time make them feel uncomfortable. In their fantasies, teen girls dream of their “prince charming”. The ending of a relationship that at first was seen as “ideal” causes loneliness and may eventually harm self-esteem. In their longing to be loved and recognized, adolescents can overlook the unpleasant traits in the person they have chosen as a partner and highlight those they consider their qualities, thus losing a sense of proportion of reality. This invokes the saying “Love is blind”. Many teens who fantasised about their “prince charming” are faced with the situation of feeling “used” after they were promised the “moon and the stars”, that is, after their partner promised them that he would be with them “for better an for worse” in an effort to convince them to have sex and then leave them. These girls sometimes become pregnant and suddenly have to face reality. It is therefore important for adolescents to more objectively observe their likely partner and reflect on their personal goals and decisions.

It has also often been argued that pregnant adolescents did not sufficiently appreciate the negative consequences of not using contraception, that they did not use any method since it was their first experience and they did not consider that they could become pregnant, and that some of them even wanted to have a child. Our results match these findings [31]. Accordingly, we believe that pregnancy prevention programs in adolescents should be directed at the participants’ attitudes towards pregnancy, since the use of effective contraceptives is shaped by such attitudes and strongly associated with the reduction of the risk of pregnancy.

### The experience of fatherhood

Once adolescent pregnancy occurs, the male must make a decision. He may choose to stay with his partner, either by forming a family with her and providing for his child in some way, or, alternatively, by removing himself from that responsibility, either due to external pressure, or as a freely made choice. While in some cases, adolescent fatherhood is a ritual of passage to adult life through which the male perceives himself as becoming a true man; it is also viewed as a disadvantage to carrying out plans and achieving. Our data showed that the boyfriend who responded to the pregnancy by living with his partner did so to live up to the stereotype of a true adult man. This implied an emotional attitude of the boyfriend towards the pregnant teenager and the consideration of the possibility of living the experience of fatherhood. This matches the findings of a study of a group of Brazilian adolescents [32] who experienced fatherhood, and in which it was concluded that, in general, men were not able to achieve their objectives in life as a result of their commitment to fatherhood.

The analysis of our interviews showed that fatherhood was accepted despite the implications of dropping out of school and getting a job, either in the informal economy or in a poorly paid job in the formal sector, the options available for job seekers with elementary and middle school educational levels. This has been demonstrated [33] in a group of men and women who became parents during adolescence and who had to drop out of school to work. The male partner jeopardized his own academic and economic future, as well as that of his child, resulting in limited future expectations for adolescent parents. In this regard, our findings are similar to those reported by Fletcher and Wolfe [34], who showed that adolescent fatherhood, results in fewer years of schooling.

The present study shows that marriage did not materialize in the vast majority of the cases analysed, which is probably due to the high costs of a civil or church wedding. Furthermore, at this stage of an adolescent male’s life, he usually depends on her family and, if he works, it is for extremely low wages. These findings match those reported from a study of a group of adolescents in the United States [34] who accepted their paternity. It showed that most teenage couples opt for common-law marriage, since, given their economic resources, they could afford to cover only part of their basic expenses.

When the teen couple lived in uxorilocal or virilocal residence, their long-term goal was to become independent because of the persistence of conflictive family dynamics. Even considering the difficulties implicit in this situation, the residence provided by the older generation was at first of great assistance. In extended families, the aid to the new couple in some cases included caring for the child as well as contributing to the housework and household expenses, which coincides with the characteristics of the Mesoamerican family system, as shown by Robichaux [35, 36] for the Mesoamerican cultural area. However, the teens living in virilocal residence described feeling a certain pressure. Some of them even commented that this situation made them feel like domestic servants in their own home, or like they were under surveillance [35]. This led to the girl breaking up with her partner or to frequent changes of residence, as Robichaux has also demonstrated in Mesoamerica [36].

### When the teenage mother goes to school

Support from the parents in order for the adolescent to attend school was found to be beneficial in this respect, it has been reported that, when a teenager attends school, her life project alternatives are very different from those of adolescents who are not in school. Opportunities are also different, and it has been shown that teens who attend school have a lower risk of becoming pregnant during this stage of life. As has also been demonstrated in a study of 1790 Mexican adolescents between 12 and 19 years old, 74.9% of the sample with a history of pregnancy had failed at least one school year, and it was shown that school failure doubled the risk of adolescent pregnancy [37]. In Our research found some evidence suggesting a relationship between educational aspirations and postponement of plans or intentions to start a family. Perception of the importance of attaining higher education levels favours having children after age of 19. This suggests the need for programs aimed at supporting adolescents in setting personal development goals in which emphasis is placed on the impact of early parenthood in thwarting such goals. The school would be the ideal setting to implement such programs. When achieving higher levels of education is not encouraged in the family setting and when resources to invest in education are lacking, children are more likely to reproduce the life trajectories of their parents.

In the developing countries, the fertility rate in the adolescent population has not diminished because there has been no substantial increase in the opportunities for access to comprehensive education or in well-paying jobs; public programs that promote the prevention of teen pregnancy have been lacking. Faced with this problem, the National Strategy for the Prevention of Adolescent Pregnancy was implemented in Mexico for the purpose of reducing the number of adolescent pregnancies with absolute respect for human rights, particularly sexual and reproductive rights, as well as to contribute to human development and expand the employment and educational opportunities of adolescents in Mexico [38]. However, this program had not yet been created at the time of our research interviews.

The literature has demonstrated that early motherhood occurs in a greater proportion among teenagers who come from a lower socioeconomic background, and that it is not the pregnancy that promotes dropping out of school, but socioeconomic limitations instead. In Mexico, of the total number of teenagers who are mothers in a situation of poverty, 95% dropped out of school before getting pregnant and only 1% of those that got pregnant were able to return to school after the child was born [23, 37, 38]. This situation was reflected in our study: all of the pregnant teens in the sample dropped out of school either before or after the pregnancy. Only four single mothers of these 29 girls could later resume their studies.

### The life of teenagers as young mothers

All the adolescent mothers interviewed in this study offered ambiguous narratives about the concept of motherhood. Our findings coincide with those reported by Tanner et al. [39], who showed that the perspectives and values of the family core group reflected traditional and expected norms for pregnancy and parenting, but these values did not always accurately reflect the values of adolescents regarding pregnancy and parenting their children.

In our study, some teen mothers mentioned that their child was an essential element in their life to avoid feeling lonely. Others thought of themselves as fulfilled regarding their conception of motherhood, but with frustrations because of dropping out of school or leaving their job. Others said that they felt limited in their social life due to the responsibility of caring for their child and attending to their partner. These ambiguities may be the result of adolescent parents’ perspectives, as well as the uncertainty and insecurity implicit in having a child at such an early age, implying economic limitations and little support from their partners.

On the other hand, the teen girls felt disappointed upon discovering that pregnancy and motherhood were not what they had imagined, due to the fact that their affective aspirations were not satisfied in the way that they had expected. All of these situations can put their emotional bond with their child to the test, who can easily become a target of their frustration and resentment. One possible explanation for this behaviour is that when they had suitable emotional and material support from their family and husband-partner they were less stressed and anguished.

It should be noted that all adolescents at the beginning of their pregnancy referred to the idea of having a family as a goal in life, without considering other development options, as was also the case in the study carried out by Tanner et al. [39]. In this sense, the adolescents we analysed sought to voluntarily start their own family to acquire adult status. This was also found by Catharine Good [40] in a group of adolescents from Guerrero, Mexico, where the majority of adolescents wished to have a family. However, life with their partner was not what they expected and they continued to feel loneliness, dissatisfaction, insecurity and, now, faced serious economic problems. This situation shows that family relationships were probably not optimal. In our reasoning in the results section, unequal relations, lack of love, and violence were the most frequent causes reported by the adolescents interviewed.

Furthermore, adolescent motherhood is a serious problem since as long as rates do not decrease and there is a high percentage of unwanted cases, it involves higher reproductive health risks than in adulthood and results in a situation of social exclusion throughout life because the majority of the cases involve poor, unwed mothers with low education levels. As shown by Kershaw et al. [41], most adolescent pregnancies are unwanted and put teen mothers in a situation of exclusion among their peers. Adolescent pregnancy negatively affects reproductive rights and gender equity [42], fostering loneliness and depression as the girl’s expectations of a family filled with love are far from actual reality. In addition, it is a type of problem with complex and multiple roots thus requiring a transversal criterion and including different levels of action.

On the other hand, there were cases in our data in which the adolescent mother preferred that her partner not live with her because of their problematic relationship. For couples living together, males generally had to work long hours outside the home, which meant that they generally had little time to spend with their partner. When the two spent time together, the relationship was often fraught with conflict, as demonstrated by Desrosiers et al. [43]; where it was concluded that evasive and anxiety-ridden forms of attachment were associated with depressive symptoms and, therefore, a conflictive relationship.

Although the adolescents in this study voluntarily formed a couple relationship, the age at which they decided to start their family was a determining factor in the stability or instability of this long-term life project. Consequently, living together or marriage among adolescents is a questionable project due to the context, including the frustration, despair and loneliness that lead to the failure of these relationships. Another factor was the limited economic possibilities of these young couples. Our findings coincide with those reported by Manning and Cohen [44], who analysed adolescent cohabitation, marriage and motherhood. The vast majority of unmarried pregnant teens did not form a union before the birth of their child; however, most pregnant teens ended up living with their partner. Cohabitation is an important part of the life of the teens, and many teenage mothers described as “single mothers” are actually in living in common-law relationships.

### Being a single mother is better?

Furthermore, the single mothers who were supported by their parents had a greater possibility of continuing their studies. This is similar to the European system of marriage, in which marriage later in life has the aim of assuring greater independence and socioeconomic level of the couple [45]. Unlike the European system of marriage, in which the prevailing type of family is nuclear, in the present study the extended family was predominant. Thus, the teen mothers with this kind of family always had a support network. However, the long-term results found in this study were quite distinct from those reported for the European system [46]. That is why early marriage and its consequences in Mexico and Latin America should be the object of more careful examination.

### Child marriage: a harmful traditional practice that violates human rights

According to Taylor’s study in Brazil [47], the main factors that lead to marriage in adolescents include: unwanted pregnancies and the interest on the part of the girl’s family in protecting her reputation of the adolescent; a desire to ensure the future of their child; the desire to control the sexuality of the adolescent, and the desire on the part of the adolescent’s family to avoid situations of shaky economic security. Another factor involved in the marriage of adolescent girls, especially in rural regions, is the desire on the part of men to marry younger wives who are considered more attractive and easier to control. In the Brazilian sample, husbands were on average 9 years older than their wives, notably contrasting with our results which showed that the girls’ partners were also minors. Furthermore, the girl’s parents did not seek to exclude her from the family, although in the majority of cases, the relationship between the father and the girl was strained.

In Latin America and the Caribbean, marriage between adolescents is more frequent in urban or suburban areas, while in rural areas of the proportion of adolescent girls marrying adult men is higher. In our data, all adolescents had a family history of teenage pregnancies and unions. This is perceived across generations as a common event in the family and the community, for the sole purpose of starting a family and, thereby attempting to cover their need for the love and affection that was not received in the family as a child.

It is can thus be concluded that this combination of factors leads to practices detrimental to the human rights of girls, boys, adolescents and women. Of particular concern are the right to health, education, a life free from violence and the right to express their opinions regarding sexual and reproductive health, all of which have an impact on their life plans. Although poverty is not the cause of child marriage, it significantly affects the life projects of girls and adolescents. To the extent that girls and adolescents do not have access to education, they have fewer opportunities to make informed decisions about actions outside the home. As a consequence, they can be seen as an economic burden by their families, even as unable to support themselves.

### Implications of the study

The present study provides information that could help decision makers reflect upon and develop adequate intervention measures to improve communication between parents and children, particularly in families that have a background of teen pregnancy. With such intervention, it would be possible to detect whether there are teen girls experiencing loneliness, uncertainty and, in such cases, intervene to try to avoid a pregnancy.

One of the implications of our findings is that adolescents could benefit by institutional programs that address their need to be listened to, and health services that protect their privacy and identity, and show a willingness to address any issue, to earn their trust, to go where they go and speak their language. Programs should be developed that cut through red tape, eliminate inefficiency, show empathy and provide anonymous attention. Although experience has hitherto been limited, programs that have worked best have involved the adults responsible for adolescents’ education and included adolescents in their design. Such programs have stressed interpersonal communication and the articulation of the school with the support services provided. Good results can be obtained when lower-risk models are presented, sufficient time is invested and a willingness is shown to attend to adolescents’ issues.

Another implication of this study is that sexuality must be be assumed as a right, but a right to be responsibly exercised in an informed manner, under conditions of autonomy and equity in which there is no place for domination, violence or imposition. To this end, adolescents need to know who they are, what they want and to recognize their own personal worth. From a platform from which they achieve a sense of definition of self and their own personal security, they can be congruent in their social behaviour, enabling them to share their qualities with their peers instead of competing against them. Achieving identity and personal autonomy is a result of breaking away from family protection and the recognition of their own needs and satisfactions. It entails the definition of one’s own values and expectations as well as transferring affection centered on their parents to other persons.

In addition, it is imperative to strengthen sexual education, not only by providing information on physiological aspects, but also through identification and satisfaction of individual needs and dealing with doubts, concerns and expectations, as well as other factors. The advantages, disadvantages, indications and contraindications should be talked over with adolescents, emphasising the correct use of condoms and other contraceptives. Parents, partners and teachers should be involved in the sexual education process and adolescents should be encouraged to participate in the development of educational models (posters, theatre, etc.). Adolescents can thus have access to the knowledge and support necessary to make responsible decisions regarding their own sexual behaviour.

Finally, it is important to consider that adolescents throughout the world experience a high percentage of unexpected or unplanned pregnancies, sexually transmitted diseases and other problems related to sexual and reproductive health. For many groups lacking services, the services offered may not be compatible with their cultural, social or economic traits, a reason for which they may not be well received. Consequently, developing strategies to provide preventive services for adolescents is a complex administrative challenge, which must address cultural differences and particular social and economic situations. Before any action can be taken, the target population and its needs, as well as the services intended to be implemented, must be identified. The objective sought to be achieved must be set and the pertinent actions to be taken must be defined. Subsequently, the service provider should apply the necessary administrative processes in order to implement, coordinate, supervise, control and evaluate the actions aimed at achieving the goal. This has implications regarding technical components, costs and administrative aspects inherent in the current system of service provision. Reports, as well as needs expressed by the population served and observed by the service provider, can recurred to in order to address this issue. In identifying needs, rapid and inexpensive methods should be used to collect both qualitative and quantitative information.

In addition, with the information obtained from the analysis, future research should be focused on male sexuality and adolescent fatherhood. This will provide us with relevant information about couple dynamics and, more importantly, new knowledge for prevention.

### Limitations of the study

One of the limitations of this research was that we have not delved into the psychological aspects of the participants. Consequently, it should be noted that the search for love may have been the result of a depressed state and low self-esteem. Where sexual intercourse was not occasional, but occurred in a love-seeking framework, the adolescents in our study reported that it took place within an environment of affection or love with their boyfriends. The question of what love was for them was still unresolved at that time, although this was not one of the aims of our research. In this regard, pregnant teenagers stated that sexual intercourse occurred within an environment of affection with her boyfriend.

Another of the limitations of the present study is its qualitative methodology and lack of external validation. Its importance centres on aspects related to the credibility, applicability and consistency of the results. Accordingly, the data may be representative of adolescents who have the same sociocultural and family context in the study as described by the interviewees, above all in the families that, out of necessity, find both parents working, and thus have no other choice but to leave their children alone or with other people.

## Conclusions

Despite teen mothers’s expectations to continue their and later obtain a good job and get married were not met, it was observed that adolescents seeking love and understanding did not prevent a pregnancy. This occurred in a specific socio-cultural framework that exhibited both social and economic limitations for teens.

It is vitally important that the family setting promotes motivation in adolescents in order for them to continue their studies. Adolescents who are more likely to attend school have lower risk of becoming pregnant, and pregnancy at an early age is not one of their life choices. It is thus necessary to encourage the creation of effective policies and strategies to prevent adolescent pregnancy while at the same time promoting greater educational and work opportunities for adolescents, in order to close the gap in schooling between adolescents with and without children.

Although there has been an increase in the average number of years of schooling in women in recent decades, the quality of basic and higher education including sexual and reproductive education remains a challenge for the Latin American and Caribbean region. It is consequently necessary to continue efforts to improve the quality of basic and secondary education and making sure adolescents remain in school system, thus favouring their successful transition to adulthood.

Dysfunctional family relationships were frequently reported by the adolescents interviewed. It can thus be inferred that a possible cause of teenage pregnancies in the present study was the perceived need of adolescents to leave a conflictive family dynamic in which they did not find support or receive motivation, and where the vast majority of conflicts stem from the father’s poor relationship with his teenage children. With respect to a family history of teen pregnancy, it is concluded that it is an important factor in the family context, and this is probably due to the fact that the socioeconomic and family environment had not changed over the last generations.

Many of the problems inherent in adolescence are related to the lack of affection and family support and may often be derived from a reaction to authoritarian rules or limits unilaterally established by parents with little or no dialogue involved. But despite the increased conflicts that usually accompany the arrival of adolescence, in most families those complicated initial moments are overcome and a new balance is struck, satisfactory to both parents and children. We believe that it is important to project a more realistic image of adolescence, removed from those topics and stereotypes that portray adolescents as conflictive, violent, and in permanent struggle against adults. When parents have such pessimistic expectations it is not surprising that they end up being fulfilled. It is important for parents to understand that while relationships with their children will change during these years, they can still be very rewarding.

Parents continue to be important, and therefore, how they relate to their teenage daughters and the parenting style they display will be of great importance for both the development of the adolescent and the emotional well-being of the parents themselves. There are a number of ideas that should be conveyed to them to assist them in the exercise of their parental role. On the one hand, parents need to know the main changes that their daughters will experience during this stage, as well as their new needs. With such information they are usually less confused and distressed and react more rationally and reflexively in face of the new behaviour patterns. But it is also important that they learn how to develop a proper parenting style, a style that combines affection, communication and support with the encouragement of autonomy and individuality.

Finally, it is important to advise parents on their parenting and parenting tasks, as this new stage may be more complicated for many parents who may feel disoriented and confused. They must be able to count on resources supportive of them in their educational task, and it is necessary to provide them with knowledge and strategies so as to increase their competence and improve their parenting style and serve to bolster their ties with the community. Regarding teen sexual relations of a romantic or social nature, it is important that the health personnel involved in work with adolescents understand the complexity of relations at this stage in life, as this is an essential factor for the success of sexual education and reproductive health programs.

In general, sexual education in Mexico and the rest of Latin America and the Caribbean has been lacking in a comprehensive vision. There is no culture of prevention from the standpoint of sexual and reproductive rights, sexuality, sexual health and gender, and the consequences of this deficiency can be observed in Mexico’s health indicators. The aim of education in the field of sexuality and reproductive health should be to promote changes in behaviour and attitudes, in pursual of the objective of communication as a process of social interaction that must provide clear and precise messages. It is for this reason there is a need to develop models of intervention that explain reality so they can generate active learning, critical awareness and ethical discernment, all in a framework of a sexual rights culture.

As is the case in other parts of Latin America and the Caribbean, our study in Mexico showed that legal and common-law marriage among children or adolescents is a frequent problem that is yet to be solved. Generally speaking, teenage couples opt for common-law living arrangements because their scarce economic resources allow them to cover only part of their basic expenses, and they cannot afford the costs of a religious or civil wedding. On the other hand, adolescents who accepted their paternity had fewer years of schooling, which reduced their work options, as well as the probability of receiving a professional degree. Their outcome compared unfavourably to adolescent mothers who did not opt for cohabitation with their boyfriend or partner and who had the support of their parents to continue their studies.

When adolescent relationships are characterized by an evasive and anxiety-filled attachment, such behaviour is usually related to depression. Given their conflictive nature, different approaches to couple relations should be considered to be able to support the transition to parenthood and the development of the necessary skills to foster the attachment and the challenges that arise in adolescence couple relations. On the other hand, secure attachments, equitable relationships, feelings of love and an absence of violence are particularly important. Adolescents need strong relationships to enhance their mental and physical health. Individual and family contexts characterised by a dynamic of open communication and emotional support may help to dissuade them from leaving the family setting in search of affection elsewhere.

## Additional file

Additional file 1: Interview guide for pregnant teens. The interview guide provided information concerning the family context and the individual situation of the teens, and it was divided in three periods, before, during and after pregnancy. (DOC 61 kb)

## Abbreviations

UNICEF: None United Nations Children’s Fund
WHO: World Health Organization
