# Supplementary material for: Family context and individual situation of teens before, during and after pregnancy in Mexico City
Source: BMC Pregnancy Childbirth. 2017 Nov 16;17:382. doi: 10.1186/s12884-017-1570-7 (PMC5689201; doi:10.1186/s12884-017-1570-7)
Supplement: Additional file 1: — Interview guide for pregnant teens. The interview guide provided information concerning the family context and the individual situation of the teens, and it was divided in three periods, before, during and after pregnancy. (DOC 61 kb) [file 12884_2017_1570_MOESM1_ESM.doc]

**Project: “Adolescent mothers’ support network”**

Interview guide for pregnant teens

Advertence

Confidentiality and consent:

Your answers are completely confidential and the information you provide will be used solely for the purpose of learning about the types of support and help that you receive from persons around you. You are free to not answer any question you wish and can end the interview at any time. We thank you for your help in carrying out this study. Your opinion is very important for us. There will be three interviews, each of which will take about 45 minutes. Are you willing to participate? We would like to record your interview in order to accurately take down your opinion or point of view on the different topics we are studying. Will you allow us to record the interview? Thank you very much for your participation.

NAME and Signature

Participant

Interviewer_____________________________________________ (Signature of interviewer and record that informed consent has been obtained to make the interview and record it).

**IDENTIFICATION SHEET**

**(REGISTER THE FOLLOWING INFORMATION IN THE FIELD DIARY AND IN THE CASSETTE)**

**Interviewee**

Place of interview

Date of interview

Approximate interview time

COMMENTS OF INTERVIEWER ON THE DEVELOPMENT OF THE INTERVIEW (Participation, information, experience of the interviewee on the topics touched upon in the interview, willingness to provide information, relevant points of the interview, difficult aspects to explore or delve into, others).

**GENERAL CHARACTERISTICS**

1. **BEFORE PREGNANCY**

I would like to start this talk with some questions about your life in general.

**AGE**

- How old are you?

**PLACE OF BIRTH**

- Where were you born? Town, county and state.

**EDUCATION**

- What was your last grade completed in school?
- If you were in school, why did you leave?

**MARITAL STATUS**

- What is your marital status?

If married, ask:

- How old were you when you married?
- How old is your husband?

In the case of an unwed mother, ask:

Did your partner propose living together at any time? If you refused, what were your reasons for refusal?

**FAMILY AND RESIDENCE**

- Who do you live with now?
- What is your role there? (Wife, daughter, daughter-in-law)
- What is your position in order of birth among your siblings? And, how many people live in your home?
- Who owns the dwelling (parents or mother- and father-in-law).

**WORK AND EARNINGS**

- What is your occupation?
- What is your usual activity?
- What jobs did you have in the last 12 months?
- Were you in school?
- Did you receive an income from your job?
- What other income did you have?
- Who provides you with economic support (husband, siblings, spouse’s parents, relative, parents) and how many people depend on this income?

**RELATIONSHIP WITH FAMILY AND PARTNER**

- What were your ties to your in-laws?
- Who supports you the most and in what situations?
- Who do you turn to when do you feel lonely and feel like talking to someone about a topic that concerns you?
- What was the relationship between you and your parents before you got pregnant?
- How was the relationship with your partner in terms of reactions and emotions, during courtship prior to the pregnancy?

**RELATIONSHIP AND SUPPORT FROM PARENTS AND SIBLINGS**

Relationship with her parents

- What was the relationship between your parents before you got pregnant?
- How would you describe the relationship with your parents over the course of your pregnancy compared to before getting pregnant?

In case she did not live with her parents

- How do you feel about the absence of your parents and what are the reasons for the absence of one, or both of them?
- Did any first-degree relative become a mother before her nineteenth birthday?
- Did any of your relatives-in-law become a mother before age 19?

**SIBLINGS**

- Do you have siblings? What is your place in terms of birth order?
- Who provided you with help, brothers or sisters?

**SEXUAL AND REPRODUCTIVE HEALTH**

- How old were you when you had your first sexual relationship? What was your relationship with this person? And, how old was that person?
- Did your partner agree to the pregnancy?
- Who made the decision to use or not to use birth control?
- Was there an abortion attempt? If so, what methods or remedies did you use for it?

**EMOTIONS**

How did you feel before your pregnancy in terms of your emotions and feelings?

- What future expectations did you have before you became pregnant? For example, studies, travel, work and marriage.
- What were the reasons why you became pregnant?

1. **DURING PREGNANCY**

**REACTIONS TO THE NEWS**

- How did your parents and family react to the news of the pregnancy?
- How did the partner react? Did he offer you help?
- How did he take responsibility to support your pregnancy?

**RELATIONSHIP WITH FAMILY AND PARTNER, REACTIONS**

- Have you noticed any changes in your partner's attitude since your pregnancy?
- What were those changes, and what do you attribute them to?
- How would you describe the relationship with your parents in the course of your pregnancy, compared to the relationship you had when you were not pregnant?
- Who do you live with now that you are pregnant? And where?
- How do you feel about your pregnancy? Mention your emotions and feelings, please.

If you have changed your residence and do not live with your parents

- For what reason did you change residence?
- How do you feel about the absence of your parents?

If your siblings or friends are with you

- How did they react to the news of your pregnancy?

1. **AFTER PREGNANCY**

**RELATIONSHIP WITH FAMILY AND PARTNER, REACTIONS**

- Are you still in contact with your partner?

After the birth of your child

- What kind of the relationship you have with your partner?

From the birth of your child

- Have you noticed any changes in your partner's attitude?
- What are these changes and what to you attribute them to?
- How would you describe your relationship with your parents now that their grandchild was born compared to the relationship you had during pregnancy?
- Who do you live with now and why?

**FEELINGS**

- How do you feel now that you are a mother?

In case of being married or single

- How do you feel as a wife or single parent?
- Which of your expectations did you have prior to pregnancy, were you able to keep up to date?
- What expectations do you have now that you are a mother?
- What persons supports it now that your baby was born?
- What is your self-perception as a woman and as a mother?

**THANK YOU FOR YOUR TIME AND PARTICIPATION**
